# Bilinguals’ Sensitivity to Grammatical Gender Cues in Russian: The Role of Cumulative Input, Proficiency, and Dominance

**DOI:** 10.3389/fpsyg.2018.01894

**Published:** 2018-10-11

**Authors:** Natalia Mitrofanova, Yulia Rodina, Olga Urek, Marit Westergaard

**Affiliations:** ^1^Department of Language and Culture, UiT The Arctic University of Norway, Tromsø, Norway; ^2^Department of Language and Literature, Norwegian University of Science and Technology, Trondheim, Norway

**Keywords:** nonce words, default gender, heritage speaker, Norwegian-Russian bilinguals, transparent/opaque gender, proficiency, cumulative length of exposure, lexical diversity

## Abstract

This paper reports on an experimental study investigating the acquisition of grammatical gender in Russian by heritage speakers living in Norway. The participants are 54 Norwegian-Russian bilingual children (4;0–10;2) as well as 107 Russian monolingual controls (3;0–7;0). Previous research has shown that grammatical gender is problematic for bilingual speakers, especially in cases where gender assignment is opaque (Polinsky, 2008; Schwartz et al., 2015; Rodina and Westergaard, 2017). Furthermore, factors such as proficiency and family type (one or two Russian-speaking parents) have been argued to be important. Interestingly, previous findings differ with respect to the kind of errors children make: restructuring to a two-gender system (masculine–feminine, see Polinsky, 2008) or defaulting to masculine (see Rodina and Westergaard, 2017). It is also not clear to what extent children are sensitive to gender cues or whether certain agreement patterns are simply memorized. To investigate this, we used both existing nouns and nonce words and tested both transparent and opaque gender cues. The results were checked against a number of background factors measuring exposure, proficiency, and dominance. Our findings show that bilingual children are clearly sensitive to morphophonological cues for gender assignment. The most common and robust error pattern for all bilinguals involved overgeneralization to masculine (especially affecting neuter and opaque nouns). At the same time, children from families with two Russian-speaking parents and monolinguals also occasionally overused feminine with vowel-final nouns. The following variables were found to be the most reliable predictors of accuracy on grammatical gender tasks: cumulative length of exposure (CLoE) and consistency of input in Russian, as well as the presence of older siblings, with CLoE to Russian being by far the most robust and important predictor. Furthermore, we show that a lexical diversity measure (number of different words in a Russian narrative) is also correlated significantly with the children’s performance on the gender tasks. At the same time, our results indicate that relative measures of dominance (e.g., the difference in exposure between the two languages or the difference in narrative scores) may be redundant when more robust absolute measures are present (CLoE and lexical diversity in the heritage language).

## Introduction

In this paper, we investigate heritage speakers’ sensitivity to gender cues in Russian through a prism of a composite measure, combining linguistic background variables as well as measures of general proficiency and dominance. This novel method allows for a more direct way of measuring the predictive power of different variables for bilinguals’ linguistic competence. The Russian three-gender system (masculine, feminine, and neuter) is relatively transparent, with some opaque cases, and it has been shown to be in place early in monolingual L1 acquisition. However, grammatical gender has been argued to be somewhat problematic for certain groups of heritage speakers, who have been found to develop a reduced gender system of only masculine and feminine (Polinsky, 2008) or no gender system at all, defaulting to masculine (Rodina and Westergaard, 2017). The factors that have been invoked to identify these groups of heritage speakers include general proficiency (Polinsky, 2008) as well as family type (one or two Russian-speaking parents) and amount of input (Rodina and Westergaard, 2017). In the current paper we use a much more detailed battery of 20 background variables as well as a proficiency measure based on semi-spontaneous narratives. In order to test whether heritage speakers are sensitive to morphophonological gender cues (and do not just memorize item-based patterns), we designed gender tasks that include both existing and nonce words. The participants for the study were 54 bilingual children growing up in Norway (age range 4;0–10;2) and 107 monolingual controls in Russia. The bilingual participants were from families with two Russian-speaking parents (the RR group) or families with one Russian- and one Norwegian-speaking parent (the NR group). The results show that, while there is considerable defaulting to masculine in the production of some of the heritage speakers, the general picture is that they are clearly sensitive to gender cues in the nonce word task. Furthermore, with respect to the background variables and proficiency measures, the statistical analysis shows that the best predictors of the children’s performance on the gender tasks are a combination of three background variables (cumulative length of exposure (CLoE), consistency of input, and the presence of an older sibling) and one proficiency measure (lexical diversity in the narrative task). We argue that this shows that language dominance in heritage speakers is a relative concept that must take a number of factors into account in order to explain the acquisition of complex linguistic phenomena such as gender.

The paper is structured as follows: In the next section, we provide some background for the study, including a brief description of the gender system of Russian, an overview of previous research on the acquisition of gender in heritage language, and a discussion of commonly used proficiency and dominance variables. Section “Research Questions and Predictions” introduces our research questions and corresponding predictions based on previous findings, and Section “Materials and Methods” provides an overview of the participants of the study, the gender tasks, the background variables collected, as well as the language proficiency measures. In Section “Results,” we present the results of the study and a detailed analysis in terms of a number of statistical models. Section “Discussion” contains a discussion of our findings and Section “Conclusion” provides a brief conclusion.

## Background

### Gender in Russian

Russian distinguishes between three grammatical genders – masculine, feminine, and neuter. Gender agreement is expressed as a suffix, and appears on singular adjectives, verbs in the past tense, demonstratives, participles, and certain pronouns. This is illustrated in (1). In the glosses, the gender of the noun is marked in parentheses and the agreeing item is marked after a full stop. In the present study, we only consider adjective-noun agreement in the nominative singular.

(1) Gender agreement marking in Russian
  a. Moja bol’šaja mašina
      My._F_ large._F_ car_(F)_
      ‘My large car’
  b. Moj bol’šoj slon
      My._M_ large._M_ elephant_(M)_
      ‘My large elephant’
  c. Mojo bol’šoje krylo
      My._N_ large._N_ wing_(N)_
      ‘My large wing’

The distribution of genders in the lexicon is uneven, with masculine nouns constituting approximately 46% of all nouns, feminines 41%, and neuters only about 13% (Corbett, 1991). Masculine is usually considered to be the default gender, since it is the most frequent, attracts most borrowings, and is associated with the default declension class (Corbett, 2007, p. 267). In addition, masculine agreement is used to refer to mixed-gender groups and in cases where the biological gender of an animate referent is unknown or unclear (Corbett, 2007, pp. 271–272).

Gender assignment in Russian is largely predictable, i.e., the grammatical gender of the noun is usually evident from its phonological shape in the nominative singular. Thus, nouns ending in non-palatal consonants are masculine (e.g., *stol* ‘table’), nouns ending in stressed [a] are predominantly feminine (e.g., *noga* ‘leg’), and nouns ending in stressed [o] are neuter (e.g., *steklo* ‘glass’). Such nouns will be referred to as transparent. However, in certain cases the form of the noun in the nominative singular is opaque. For example, both feminine and masculine nouns may end in palatal and postalveolar consonants in the nominative singular (e.g., *gus*’ ‘goose.MASC,’ *rys*’ ‘lynx.FEM’). Gender marking on nouns ending in palatalized consonants has been found to be problematic in monolingual first language acquisition, where overgeneralization to the masculine has been observed with feminine nouns during the preschool years (Gvozdev, 1961 based on diary data; Ceitlin, 2005, 2009 based on corpus data). This is likely due to the higher frequency of masculine nouns. It should be noted that the opposite, i.e., using feminine forms with masculine nouns ending in palatal consonants has not been attested in monolingual children. Other non-transparent nouns include those ending in unstressed vowels. Due to the application of a vowel reduction process, underlying vowels /a/ and /o/ both get realized as [ǝ] in unstressed position, making nouns like *part[ǝ]* (‘desk.FEM’) and *sit[ǝ]* (‘sieve.NEUT’) opaque with respect to gender (see Iosad, 2012 on vowel reduction in Russian). Russian children have been shown to overgeneralize feminine agreement with non-transparent neuter nouns (Gvozdev, 1961; Popova, 1973). The opposite pattern, i.e., neuter agreement with stem-stressed feminines, has not been attested. All phonologically opaque nouns can be disambiguated by the case paradigm that they follow (e.g., *gus*’*-u* ‘goose-MASC.DAT’ vs. *rys*’*-i* ‘lynx-FEM.DAT’). Thus, knowing the correlation between declensional class and gender is crucial in order to successfully predict the gender of these nouns.

Importantly, in monolingual acquisition, the masculine–feminine distinction is established very early, at approximately the age of 2 (Gvozdev, 1961; Ceitlin, 2005, 2009). Before their second birthday some children are reported to go through a short stage when feminine agreement is overgeneralized with masculine and neuter nouns (Gvozdev, 1961; Popova, 1973; Zakharova, 1973). Acquisition of neuter seems comparatively more difficult, which can be attributed to its low frequency in the input. While gender agreement with transparent neuters is usually mastered between 3;0 and 4;0 years of age, opaque neuters remain problematic until approximately the age of 6;0 (Gvozdev, 1961; Ceitlin, 2009).

The next section shows that gender marking has been found to be problematic for speakers of Russian as a heritage language. However, their overgeneralization patterns do not always match those of monolinguals.

### Gender Acquisition in Heritage Russian

Grammatical gender has been shown to be vulnerable in Russian heritage language, where both quantitative and qualitative differences have been observed in child and adult heritage speakers (e.g., Polinsky, 2008; Schwartz et al., 2015; Rodina and Westergaard, 2017). Non-target-like performance is mainly attributed to a combination of factors such as non-transparency of gender cues and insufficient exposure. To assess the role of participants’ background, studies have typically employed different measures for children and adults. While the adults in Polinsky (2008) were assessed using a range of measures including a personal history questionnaire, a lexical translation task, and speech rate in oral narratives, in studies with children, family type and parental background questionnaires have been central (e.g., Gathercole and Thomas, 2005; Unsworth et al., 2014; Fhlannchadha and Hickey, 2017; Rodina and Westergaard, 2017). At the same time, specific domain knowledge is captured by custom-tailored experiments investigating gender marking with different subclasses of nouns.

Polinsky (2008) used a combination of production and comprehension tasks with Russian-speaking adults who assigned and judged gender marking on adjectives and possessive pronouns. The stimuli included 122 inanimate Russian nouns. Interestingly, language dominance and proficiency were introduced as different concepts in the study. All heritage speakers were defined as English-dominant simply based on the fact that they lived in the United States and English was the language of the society. Yet, they had varying proficiency in Russian as measured by their speech rate in oral narratives and lexical access on a lexical translation task. The heritage speakers’ performance on the gender tasks was found to correlate with their language proficiency. Heritage speakers with faster speech rates and lexical access, defined as high-proficiency speakers, had developed a target-like three-gender system of masculine, feminine, and neuter. In contrast, low-proficiency heritage speakers developed a reduced two-gender system of masculine and feminine, as they assimilated opaque as well as transparent neuter nouns to the feminine. Polinsky emphasizes that, while the observed restructuring was found to correlate with speech rate and lexical access, it did not correlate with a distinction proposed by Au and Romo (1997), whereby participants are divided into overhearers, intermediate, and more advanced speakers based on personal history questionnaires.

Studies investigating grammatical gender in child bilinguals are more numerous, with evidence obtained in different socio-cultural contexts. Schwartz et al. (2015) studied the development of gender agreement in 70 sequential bilinguals aged 4–5 acquiring Russian in the United States, Finland, Germany, and Israel. Based on parental reports, the children across these groups were argued to be Russian dominant at the age of testing, since they were born in families with two Russian immigrant parents and entered bilingual preschools around age 2–3. The knowledge of grammatical gender agreement between adjectives and head nouns was tested with the same elicitation procedure in all groups of bilinguals as well as younger (3- to 4-year-old) and older (4- to 5-year-old) monolinguals. The stimuli included 70 Russian nouns. The analysis of the children’s errors did not reveal any qualitative differences between any of the bilingual groups and the monolinguals. However, the comparison of bilinguals with age-matched monolinguals revealed that the errors were more persistent in bilinguals, especially with feminine nouns ending in a palatalized consonant and with stem-stressed neuters. Thus, the acquisition of gender is delayed in these sequential bilinguals, even though they were classified as Russian-dominant. Schwartz et al. (2015) also suggest that the presence of the grammatical category gender in both languages of a bilingual facilitates acquisition, pointing out that the German-Russian and Hebrew-Russian bilinguals, whose majority language has grammatical gender, outperformed the English-Russian and Finnish-Russian bilinguals, whose majority language has no gender category.

Rodina and Westergaard (2017) investigated gender marking on adjectives in 20 simultaneous Norwegian-Russian bilinguals aged 4;1–7;11. The stimuli of the elicited production task included 30 Russian nouns. Bilingual family type was used as the main predictor variable in the study, since 10 children were from Russian-immigrant families and 10 children were from mixed Norwegian-Russian families. Importantly, the major difference in gender marking was found in a subset of five children from Norwegian-Russian families whose input in Russian was defined as very limited and inconsistent or mixed, since the children’s Russian-speaking mothers reported using both languages and predominantly Norwegian with their children. In the gender elicitation task, this subset of children used masculine agreement almost exclusively across all classes of nouns. The authors proposed that these children may be developing a variety of Russian with a more extensive reduction of the gender system (affecting both feminine and neuter, resulting in a system without gender), in contrast to the adults in Polinsky (2008), who showed signs of reduction of the neuter only. Like Polinsky (2008), Rodina and Westergaard (2017) suggested that this qualitative difference between monolinguals and heritage children could be due to the latter not having mastered the relatively complex declension system of Russian. These learners may thus be insensitive to the gender cues. The analysis of the bilingual data was based on two additional input measures – CLoE and the percentage of exposure at present (cf., the Bilingual Language Experience Calculator, Unsworth, 2013). Only CLoE was found to be a significant predictor of the bilingual children’s gender marking in Russian, while the children’s chronological age was the only significant predictor for gender accuracy in their majority language, Norwegian. This result was argued to support the conclusion that the amount of exposure was crucial for successful gender acquisition and that early exposure was not a sufficient condition.

Urek et al. (unpublished) used the procedure in Rodina and Westergaard (2017) to investigate gender acquisition in Latvian-Russian preschoolers resident in Riga, Latvia (*N* = 20, aged 4;0–6;10). In contrast to Rodina and Westergaard (2017), all the participants in this study come from mixed families, where one parent was a native speaker of the majority language and one a native speaker of the minority language. Crucially, the participants in this study reside in a country with a high degree of societal bilingualism and are therefore not heritage speakers of Russian *per se*. It was found that while the bilingual participants were less accurate in gender assignment than age-matched monolingual controls, they showed no evidence of restructuring or loss of the three-way gender contrast. However, just as in Rodina and Westergaard (2017), CLoE (controlling for age) was also found to be a significant predictor of accuracy.

### Assessment of Linguistic Proficiency, Input, and Dominance in Bilingual Acquisition

Bilingual speakers are a heterogeneous population, which is not surprising given that the input that children receive in the two languages can vary dramatically in terms of relative quantity, quality, and context (Sorace, 2005; De Houwer, 2007; De Cat and Serratrice, 2017). Apart from biographical variables such as the age of acquisition, chronological age, and place of birth, various measures have been proposed to quantify the amount of input that children receive, such as e.g., current amount of exposure (at home and at school etc.; cf., Gathercole and Thomas, 2009; Chondrogianni and Marinis, 2011), CLoE over time (Gutiérrez-Clellen and Kreiter, 2003; Blom, 2010; Unsworth, 2013), as well as richness and consistency of the input (Place and Hoff, 2011). Additional factors, such as the presence of siblings and birth order, language status (majority/minority) and language prestige (high/low), daycare/school type (bilingual/monolingual/immersion), friends, literacy and literacy-related activities have also been shown to affect the linguistic development of bilingual children on a par with more general exposure variables (see Unsworth, 2013, 2015 and references therein). At the same time, several studies have highlighted correlations between the following so-called child-internal factors: the amount of output, MLU, vocabulary size, children’s developing grammatical and phonological skills, fluency, and processing speed (Bohman et al., 2010; Paradis, 2011; Bedore et al., 2012).

Many of the aforementioned factors have been invoked in the discussion of dominance in bilinguals, and specifically of how dominance should best be measured. In many studies, the dominant language of a bilingual child is assumed to be the majority language of the wider community/country of residence (cf., Polinsky, 2008; see however, Schmeißer et al., 2015 for contrasting results). Alternatively, as argued by Unsworth (2015), current amount of exposure may be taken as a proxy for dominance/relative proficiency, while Treffers-Daller and Korybski (2015) propose that lexical diversity measures fit well as a means to operationalize dominance. Paradis et al. (2007) and Blom (2010) also take amount of input as the basis for determining the dominant language of a bilingual child, but also consider length of exposure since birth and amount of exposure in the home and at daycare/preschool/kindergarten. Bedore et al. (2012) apply a combination score of current language usage (current amount of exposure combined with children’s own language output) as a proxy for dominance. Finally, Montrul (2015) argues for a more holistic, multidimensional approach to dominance, which includes all the three main components: biographical variables, proficiency, and input and use factors.

It should be noted that although language dominance and language proficiency are interrelated, they are nevertheless independent parameters. For example, while the dominance profiles may be similar in two groups of speakers, their absolute proficiency in the two languages may differ significantly (as is the case of e.g., Spanish L2 learners as compared to Spanish heritage speakers in the United States, see Montrul, 2015). Furthermore, as demonstrated by Schmeißer et al. (2015), high proficiency in a language does not imply that this language will necessarily be the dominant language for a bilingual child. Moreover, language dominance is not decisive when it comes to grammatical development, specifically cross-linguistic influence. As the authors argue, absolute rather than relative proficiency in the influenced language and the degree of complexity of the linguistic construction are much better predictors of cross-linguistic influence. Furthermore, contra what is commonly believed, the language of the country of residence does not always become the dominant language of a bilingual child, and the one-parent-one-language strategy is neither a necessary nor a sufficient prerequisite for balanced bilingualism. As the authors conclude, “more research on sociolinguistic factors, external to the child, which have been neglected in the past, is needed in order to help formulate recommendations for parents, doctors, and teachers, on how to promote high proficiency levels in the two languages of a bilingual” (Schmeißer et al., 2015, p. 64).

Following this line of research, the overarching goal of our current study is to investigate in detail the relative importance of the aforementioned factors for bilingual children’s (rate of) grammatical acquisition in their minority language, specifically the acquisition of grammatical gender. Three groups of factors will be considered: language-internal factors (transparency of cues), child-external factors (e.g., current vs. cumulative exposure, relative difference in exposure between the two languages, parental language strategies, presence of siblings, etc.) as well as child-internal factors (children’s performance skills on a narrative task, as well as the difference in their performance skills in the two languages).

## Research Questions and Predictions

The present study examines bilingual Norwegian-Russian children’s sensitivity to morphophonological gender cues in Russian, their minority language. In contrast to the previous studies reviewed in Section “Background,” our experimental tasks employ both existing as well as novel nouns. This approach allows us to explore what mechanisms bilingual speakers use to assign gender and whether they develop a system of formal gender assignment rules. We also investigate the relationship between the bilinguals’ knowledge of gender and background variables such as language exposure and language proficiency. The study addresses the following main research questions:

(1) Do heritage speakers (HSs) of Russian differ significantly from monolinguals and in what conditions (on the real and nonce word tasks)?
(2) Are there any differences between nouns with transparent and nouns with non-transparent morphophonological gender cues, for both monolinguals and HSs (on the real and nonce word tasks)?

Furthermore, by comparing the results of the real and nonce word tasks we aim to answer the following questions:

(3) Do L1 children and HSs rely on their lexical knowledge of grammatical gender (i.e., is ‘accuracy’ on the real word task significantly higher or comparable with the accuracy on the nonce word task in transparent conditions, i.e., in cases where the phonological form of the noun straightforwardly predicts its gender)?
(4) Is the reliance on lexically stored gender features stronger for HSs than for the L1 children (i.e., is there a significant interaction of task and group)?

One of the main purposes of our study is to consider in detail the background of the bilingual participants. We ask the following question:

(5) Which background variables are the most reliable and robust in predicting children’s performance on grammatical gender tasks?

In addition to the background variables, we assess the value of proficiency measures (narratives) in predicting HSs’ performance on the gender assignment tasks. With respect to the contribution of the narrative proficiency measures we ask the following questions:

(6) Do narrative proficiency variables such as lexical diversity correlate with HSs’ performance on the gender assignment tasks?
(7) Do these variables help to better predict the children’s performance on the gender assignment tasks when used in combination with the background variables (i.e., is a model involving both proficiency and background variables statistically better at predicting the children’s performance than a model involving only background variables)?

Finally, we ask whether dominance variables (operationalized as the difference in exposure to the two languages and the difference in proficiency on the narrative tasks between the two languages) can account for some part of the variance observed in the children’s responses.

(8) Do variables that quantify dominance help to better predict the children’s performance on gender assignment tasks when used in combination with absolute exposure and proficiency variables (i.e., is a model involving both dominance and absolute exposure and proficiency variables statistically better at predicting the children’s performance than a model involving only absolute exposure or proficiency variables)?

Based on the previous literature on bilingual language acquisition and the acquisition of gender in heritage Russian, we formulate the following predictions:

(A) Based on the results of Polinsky (2008) we expect that bilingual children will significantly overgeneralize masculine agreement with nouns ending in a consonant and feminine agreement with nouns ending in a vowel.
(B) Based on the results of Rodina and Westergaard (2017) we predict masculine overgeneralization to be the most pervasive error in the responses of bilingual children across all conditions. We also expect family type (two Russian-speaking parents vs. one Russian-speaking parent) to be a highly significant predictor of children’s performance.
(C) We expect that the amount of input will be a highly significant predictor of children’s performance on the gender task. Following Paradis et al. (2007), Blom (2010), and Unsworth (2015), we expect CLoE and current exposure to be significant predictors of children’s performance.
(D) We expect that children’s performance on the narrative tasks will correlate with their performance on the gender assignment tasks (cf., Montrul, 2015; Treffers-Daller and Korybski, 2015 for examples of correlations between lexical and grammatical abilities of bilinguals). We assume that the acquisition of nominal gender features is based on the observation of nominal declension paradigms and agreement patterns, as well as generalizations over groups of nouns with shared morphological and phonological features. Thus, we predict that the children’s lexical diversity scores will correlate positively with their performance on the real and nonce word tasks.
(E) Finally, based on Schmeißer et al. (2015) we expect that absolute measures of children’s exposure to Russian and their proficiency scores in Russian narratives will be better predictors of their performance on Russian gender assignment tasks than variables representing relative dominance (i.e., the difference in exposure to Russian and Norwegian and differences in the proficiency measures based on Russian and Norwegian narratives).

## Materials and Methods

### Participants

For this study, we recruited 54 bilingual Russian-Norwegian children (*N* boys = 27) resident in Norway, ranging in age from 4;0 to 10;2 (mean age = 6;9). Of these, 22 children attend kindergarten, while the rest are schoolchildren. All participants in our study have a Russian-speaking mother and differ with respect to the first language of the father: 28 children (age range 4;3–9;9, mean age = 6;9) come from families with Norwegian-speaking fathers (and will be referred to as the NR group), while 26 children (age range 4;0–10;2, mean age = 6;9) come from families where both parents are Russian speakers (the RR group). All children included in this study were either born in Norway or arrived in Norway before the age of three. All come from middle-class households, where the education of the majority of the parents is at the level of an undergraduate degree. The bilingual participants were recruited and tested at Russian clubs in Oslo and Tromsø. These clubs offer weekly meetings for Russian-speaking children and provide classes on Russian language and culture (taught in Russian), as well as an informal socializing platform for Russian-speaking children and their families.

In addition, a group of monolingual controls (*N* = 107) ranging in age between 3;0 and 7;0 years (mean age = 5;2) were recruited and tested in Moscow and Ivanovo, Russia. All the monolingual children attended kindergarten at the time of testing.

### Gender Assignment Tasks

To examine bilingual Norwegian-Russian children’s sensitivity to morphophonological gender cues in Russian we used two production tasks eliciting adjectival agreement with real nouns (Experiment 1) and nonce nouns (Experiment 2). The procedure used in both experiments was an adapted version of the picture-based elicitation task from Rodina and Westergaard (2013, 2017). The elicitation materials consisted of two sets of colored pictures. The pictures used in the real-word experiment (Experiment 1) were obtained from the Colourbox database; the pictures used in the nonce-word experiment (Experiment 2) were selected from the set of pictures of novel objects included in the Novel Object and Unusual Name Database (NOUN; Horst and Hout, 2016). The pictures used in Experiment 2 all depicted inanimate countable objects of variable shapes and textures.

The stimuli used in Experiment 1 consisted of 30 picturable nouns denoting everyday objects and animals assumed to be familiar to children at the relevant age. The nouns were evenly distributed across the three genders. In addition, the nouns of each gender varied with respect to morphophonological transparency, resulting in six conditions. Examples of the stimuli are given in **Table 1**.

**Table 1:** Real noun stimuli.

Condition	M-transparent	F-transparent	N-transparent	M-opaque	F-opaque	N-opaque
Example	most ‘bridge’	lisá ‘fox’	vedró ‘bucket’	gus’ ‘goose’	kost’ ‘bone’	myl[*ǝ*] ‘soap’

The stimuli used in Experiment 2 were 25 novel nouns constructed to conform to Russian phonotactics. In order to avoid neighborhood density effects, only nouns that had no nominal phonological neighbors were selected. To achieve this, we used the Phonological Corpus Tools software (PCT, Hall et al., 2016) to check for any minimal pairs with the nouns included into the Frequency Dictionary of Russian (Sharoff, 2002). The novel nouns were equally distributed across five conditions, illustrated in **Table 2**. M-transparent, F-transparent, and N-transparent contained nouns with transparent masculine, feminine, and neuter cues respectively. The F/N-opaque condition contained stem-stressed vowel-final nouns (recall that in Russian these are ambiguous between feminine and neuter). The F/M-opaque condition contained nouns ending in palatal consonants (ambiguous between feminine and masculine).

**Table 2:** Novel noun stimuli.

Condition	M-transparent	F-transparent	N-transparent	F/N-opaque	F/M-opaque
Example	punip	kluvá	garpó	prúz[*ǝ*]	dron’

In both experiments, two pictures of the same object differing in color were presented side by side on a laptop screen. The experimenter named the depicted object and then asked the participant to name the two objects along with their colors. The experimenter then pressed a button causing one of the pictures to disappear and asked the participant to identify the object that disappeared. Thus, three instances of adjectival agreement were elicited for each target noun. Lead-in sentences were formulated in such a way as to avoid providing cues to the grammatical gender of the target noun. To familiarize the participants with the task, the test trials were preceded by two practice trials in both experiments. During the practice trials, plural forms were used to avoid priming. The elicitation procedure with a nonce stimulus noun is illustrated in (2):

(2) Elicitation procedure
  E.: Eto nazyvaetsja *punip*. Posmotri, čto zdes’?
      “This is called *punip*. Look, what is here?”
  C.: Eto krasnyj *punip*, a eto goluboj *punip*.
      this red._M_ *punip*_(M)_, and this blue._M_ *punip*_(M)_.
      “This is a red *punip*, and this is a blue *punip.*”
  E: Čto sejčas propalo?
      “What has disappeared now?”
  C: Krasnyj *punip*.
      red._M_ *punip*_(M)_
      “The red *punip.*”

All participants were tested individually by an experimenter who is a native speaker of Russian. The responses were audio-recorded and later transcribed and coded by the authors of this study.

### Background Variables

Background variables for the bilingual participants were obtained with the help of the Bilingual Language Experience Calculator (BiLEC, Unsworth, 2013), a parental questionnaire containing a set of questions designed to elicit detailed biographical data and information pertaining to the present language environment of a multilingual child in both languages, including exposure, context and use, as well as the child’s linguistic experience from the onset of acquisition. BiLEC maps, *inter alia*, the proportion of input the child receives in each of the languages (both inside and outside of the home), the proportion of the child’s own production in the L1 and the L2, and language exposure during holidays. It also includes questions on perceived receptive and productive language proficiency of the child and other members of the household (as reported by the respondent). BiLEC comes with an algorithm that automatically calculates numeric values for a range of pre-determined variables.

In the standard procedure, BiLEC serves as the basis for a parental interview. However, for the purpose of this study, BiLEC was translated into Russian and adapted into a questionnaire format in order to simplify data collection. The BiLEC questionnaires were filled out individually by one of each participant’s parents (typically the mother). The responses were then entered into the BiLEC algorithm, and the values for a range of background variables were obtained.

The variables selected for the statistical analysis fall into three broad categories: biographic variables, language exposure, context and use variables, as well as maternal input variables. The biographic variables include age in months, family type (NR or RR), group (daycare or school), place of residence (Tromsø or Oslo), and the presence of siblings (younger and older).

The numeric values for the exposure variables were calculated automatically by the BiLEC algorithm (see Unsworth, 2013 for a detailed explanation of the calculations). Traditional length of exposure to Russian and Norwegian was calculated as the time elapsed from the date of first exposure to the date of testing. Thus, the traditional length of exposure to Russian corresponded to chronological age for all the children in our sample, while the length of exposure to Norwegian only corresponded to chronological age in children coming from NR families and varied for RR children (usually depending on when the child started attending daycare). Present weekly exposure to Russian/Norwegian was calculated as a proportion determined by dividing the total number of hours per week with exposure to Russian/Norwegian by the total number of waking hours each week. We included both ‘present exposure at home’ (only taking into account the proportion of Russian/Norwegian the child was exposed to in the household) and ‘overall present exposure’ (taking into account the overall weekly proportion of Russian/Norwegian the child was exposed to at home, school, and out-of-school activities including holidays). CLoE to Russian/Norwegian (in years) was calculated as the sum of proportions of each year in the child’s life so far that included exposure to Russian/Norwegian. This measure takes into account how much each member of the household spoke each of the languages to the child during each year of the child’s life so far, the amount of Russian/Norwegian spoken at the daycare/school the child attended, and the amount of Russian/Norwegian encountered during holidays.

In addition, three variables characterizing maternal language input were considered: consistency of input in Russian (binary variable indicating whether or not the mother reported using exclusively Russian when speaking to the child), proportion of Russian input from the mother (numeric variable estimated by the parent), and maternal productive proficiency in Norwegian (self-reported using a 6-point scale from 0 ‘do not speak at all’ to 5 ‘native-like productive proficiency’).

### Language Proficiency

Language proficiency was assessed in both Russian and Norwegian for a subset of bilingual children in our sample (*N* = 27). We used the Multilingual Assessment Instrument for Narratives (MAIN, Gagarina et al., 2012) to elicit semi-spontaneous production samples. MAIN is a picture-based tool which contains four parallel stories (“Cat,” “Dog,” “Baby birds,” and “Baby goats”), each illustrated with a six-picture sequence. MAIN was chosen for the present study since it is highly suitable for the elicitation of semi-spontaneous production samples in both of the languages of bilingual children, especially between the ages of 4 and 10.

We used the model story procedure to collect production samples in Norwegian and Russian (cf., Rodina, 2017). The child first heard a pre-recorded model story while looking at the picture sequence “Cat” or “Dog” and then answered 10 comprehension questions listed in the MAIN manual. This was done in order to establish contact with the child and to provide an example of narrative production. The child was then asked to narrate a new story, either “Baby birds” or “Baby goats.” All the bilingual participants were tested in Russian first. Norwegian samples were collected approximately 2 weeks later. “Cat” and “Baby birds” scenarios were used to collect Russian narratives, and “Dog” and “Baby goats” scenarios were used for Norwegian narratives. The children were tested by research assistants who were native speakers of the respective languages. The children were tested individually, and their responses were audio-recorded and later orthographically transcribed.

In the analysis, we included two lexical measures of proficiency in each language sample: total number of words (i.e., all word tokens, TNW) and number of different words (i.e., word types, NDW). Mazes, repetitions, and incomplete utterances were excluded from the analysis. Both TNW and NDW have been shown to be important predictors of language development across different studies, including a previous investigation of narrative abilities in Norwegian-Russian bilingual preschoolers (Rodina, 2017).

## Results

We start by presenting the results of Experiment 1 (real words, subsection “Experiment 1: Real Words”) and Experiment 2 (nonce words, subsection “Experiment 2: Nonce Words”). In subsection “Background Variables,” we summarize the effects of various background and proficiency variables on the children’s performance on the gender assignment tasks.

### Experiment 1: Real Words

**FIGURE 1:**
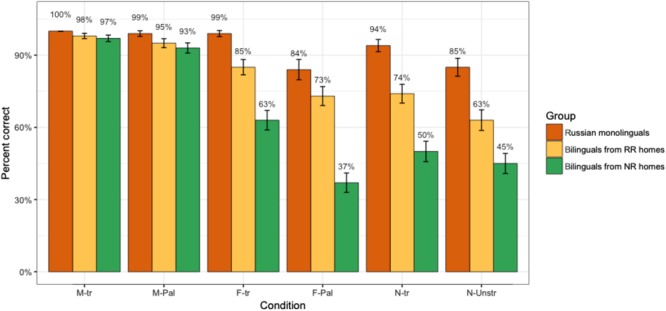
Experiment 1: Real words. Percentage of accurate responses per condition and group. **M**-**tr** – words with a transparent masculine cue, **M-Pal** – words with an opaque masculine cue, **F**-**tr** – words with a transparent feminine cue, **F-Pal** – words with an opaque feminine cue, **N**-**tr** – words with a transparent neuter cue, **N-Unstr** – words with an opaque neuter cue.

**Figure 1** presents the accuracy in gender marking across the six experimental conditions (**Table 1**) and three participant groups: Russian monolingual children, bilingual children from RR homes and bilingual children from NR homes. The accuracy rates of Russian monolinguals reveal that gender assignment is at-ceiling in M-transparent, F-transparent, and N-transparent as well as M-opaque conditions. Some non-target-like performance is observed in F-opaque and N-opaque conditions, where the accuracy rates are 85% and 86% respectively. Bilinguals from RR homes appear to be a close match to the monolinguals: F-opaque and N-opaque conditions are at 77% and 68% accuracy. However, some errors are found in the N-transparent condition, where the accuracy is 80%. Bilinguals from NR homes behave at-ceiling only in the M-transparent and M-opaque conditions. Their accuracy rates in all other conditions are below 60%.

We fit a generalized linear mixed logistic regression model where the binary variable accuracy was predicted by the interaction of Condition and Family (RR vs. NR vs. Monolingual R). Participants and items were included as random intercepts. To compare the groups within conditions, we conducted *post hoc* pairwise comparisons with the help of the R^1^ package *lsmeans* (Lenth, 2016).

*Post hoc* pairwise comparisons of the groups within conditions revealed the following contrasts:

(1) In the M-transparent and M-palatal conditions, there were no significant differences between the groups.
(2) In the F-transparent and F-palatal conditions, the NR children were significantly less accurate than the RR children (*p* = 0.002 and *p* < 0.001, respectively) and monolinguals (*p* < 0.001 in both conditions).
(3) In the N-transparent condition, the NR group performed significantly less accurately than the RR children and monolingual controls (*p* < 0.01 in both cases).
(4) In the N-unstressed condition, the RR group patterned with the monolingual controls, while the NR group performed significantly less accurately (NR vs. Monolinguals: *p* = 0.01; NR vs. RR: *p* = 0.03).

*Post hoc* comparisons of different conditions within groups revealed the following contrasts:

(1) Monolingual children were (a) significantly more accurate on M-transparent and M-palatal than on N-unstressed (*p* < 0.001 in both cases) and N-transparent conditions (*p* < 0.001 in both cases); (b) significantly less accurate on the F-palatal than on the M-palatal condition (*p* = 0.01) and marginally less accurate on the F-palatal than on the M-transparent condition (*p* = 0.08); (c) significantly more accurate on the F-transparent condition than on the N-transparent and Neuter-unstressed conditions (*p* = 0.002 and *p* < 0.001, respectively) and on the Feminine-palatal condition (*p* = 0.03).
(2) For the RR group, performance on the M-palatal condition was significantly better than on the F-palatal, N-transparent and N-unstressed conditions (*p* < 0.001 in all cases) as well as the F-transparent condition (*p* = 0.03). They were also significantly more accurate on the M-palatal condition than on the F-palatal, N-transparent and N-unstressed conditions (*p* < 0.001 in all cases). Finally, they were significantly more target-like with respect to F-transparent nouns than F-palatal nouns (*p* = 0.05).
(3) For the NR group, performance on M-transparent and M-palatal conditions was significantly more accurate than on all other conditions (*p* < 0.001 in all cases). Accuracy on the F-transparent condition was significantly higher than on the F-palatal (*p* < 0.001) and N-unstressed (*p* = 0.002) conditions.
(4) No other differences were significant.

**FIGURE 2:**
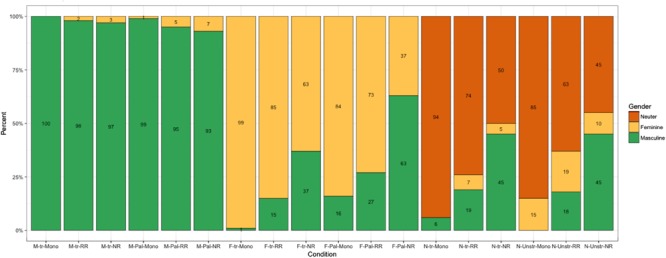
Experiment 1: Real words. The use of masculine, feminine and neuter agreement per condition (in %): **M**-**tr** – words with a transparent masculine cue, **M-Pal** – words with an opaque masculine cue, **F**-**tr** – words with a transparent feminine cue, **F-Pal** – words with an opaque feminine cue, **N**-**tr** – words with a transparent neuter cue, **N-Unstr** – words with an opaque neuter cue. **Mono** – monolingual Russian children, **RR** – bilingual Norwegian-Russian children from families with two Russian-speaking parents, **NR** – bilingual Norwegian-Russian children from families with one Russian-speaking parent.

**Figure 2** illustrates the use of masculine, feminine, and neuter agreement across all conditions and participant groups. The most common overgeneralization pattern in bilinguals involves the overuse of masculine agreement in all non-masculine conditions (F-opaque, F-transparent, N-opaque, N-transparent). This pattern is significantly more pronounced in the NR group than in the RR group. The NR group resorts to masculine across all non-masculine conditions (between 42% and 65% of the time), while the RR group overuses masculine significantly less (between 11% and 23% of the time) across all non-masculine conditions. Monolinguals erroneously use masculine 11% of the time, and only in the F-opaque condition, which bears an ambiguous feminine/masculine cue (final palatal consonant). In the N-opaque condition, where the phonological cue on the noun is ambiguous between feminine and neuter (final unstressed vowel), monolinguals overuse feminine (14% of the time), NR resort to masculine (in 51% of their responses), while RR children show both patterns (use feminine in 12% and masculine in 25% of the cases).

To sum up, in the real word experiment, we observe that the NR bilinguals are significantly different from Russian monolinguals and RR bilinguals. For all participant groups, the M-transparent and M-opaque conditions are unproblematic, while the F-opaque and the N-opaque conditions pose the most difficulty.

### Experiment 2: Nonce Words

Recall from Section “Gender Assignment Tasks” that the nonce word experiment had five experimental conditions. In the three transparent conditions (M, F, N) we expected the use of masculine, feminine, and neuter agreement. In the opaque condition, two agreement options were possible: masculine and feminine in the FM condition and feminine and neuter in the FN condition.

**FIGURE 3:**
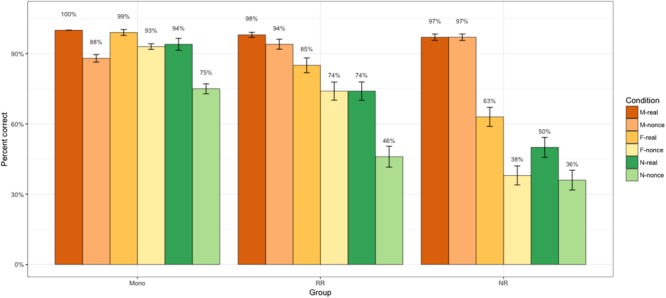
Experiments 1 and 2: Gender assignment in accordance with transparent masculine, feminine, and neuter morphonological cues on real and nonce word tasks.

We first present the results for the M-, F-, and N-transparent conditions in **Figure 3**, which compares the performance of all participant groups across these conditions in the nonce and real word tasks. **Figure 3** shows that in the three transparent conditions, children from all groups assign gender more ‘accurately’ (i.e., in accordance with the respective morpho-phonological cues) to real words than to nonce words. A generalized linear mixed effects regression analysis reveals that the ‘accuracy’ with feminine and neuter nouns is significantly higher in the real word task than in the nonce word task for all three groups of participants. Children use more masculine agreement in non-masculine conditions in the nonce-word task than in the real-word task. No significant interaction of Task and Group was found.

**FIGURE 4:**
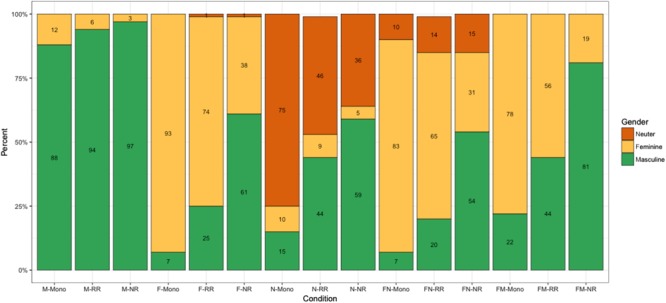
Experiment 2: Nonce words. The use of masculine, feminine and neuter agreement per condition (in %): **M** – words with a transparent masculine cue, **F** – words with a transparent feminine cue, **N** – words with a transparent neuter cue, **FN** – words with an opaque feminine/neuter cue, **FM** – words with an opaque masculine/feminine cue. **Mono** – monolingual Russian children; **RR** – bilingual Norwegian-Russian children from families with two Russian-speaking parents; **NR** – bilingual Norwegian-Russian children from families with one Russian-speaking parent.

**Figure 4** illustrates the use of agreement in the nonce word experiment in all conditions. As **Figure 4** shows, the most common overgeneralization pattern observed in the bilingual groups is the overuse of masculine in all non-masculine conditions (similarly to the real word task). Notice also that the N-transparent condition turned out to be quite problematic for the NR and RR groups. Children from these two groups produced neuter agreement in 32% and 48% of the cases, respectively, while monolinguals assigned neuter in 75% of the cases.

To analyze the differences between the groups, and more specifically, between NR and RR children in comparison with the monolingual controls, we fit a generalized linear mixed logistic regression model to predict the probability of using masculine agreement by the interaction of Condition and Family. Participants and Items were included as random intercepts.

*Post hoc* pairwise comparisons of the performance of the groups within conditions revealed the following contrasts.

(1) In the M-transparent condition, the NR group differed significantly from the Russian monolingual group (*p* = 0.003).^2^
(2) In the F-transparent condition, all groups differ significantly from each other, with the most significant contrasts being between the NR group and the monolinguals (RR vs. Monolinguals: *p* = 0.02, NR vs. Monolinguals: *p* < 0.0001, NR vs. RR: *p* < 0.001).
(3) In the N-transparent condition, the RR and NR bilingual groups differed significantly from the monolingual controls (*p* = 0.007 and *p* < 0.0001, respectively).
(4) In the two opaque conditions (F/N and F/M conditions), the RR group patterned with the monolingual controls, while the NR group used significantly more masculine than the two other groups (*p* < 0.001 for all contrasts).
(5) With respect to the agreement patterns, RR children performed similarly to monolinguals in that they preferred feminine in these conditions (83% and 69% feminine in monolingual and RR groups in the F/N condition, and 78% and 61% feminine in the F/M condition, respectively). On the other hand, NR children preferred masculine in both F/N and F/M conditions (57% and 83% masculine, respectively), while feminine was not the most frequent choice (28% and 17%, respectively).
(6) No other differences were significant.

### Background Variables

**FIGURE 5:**
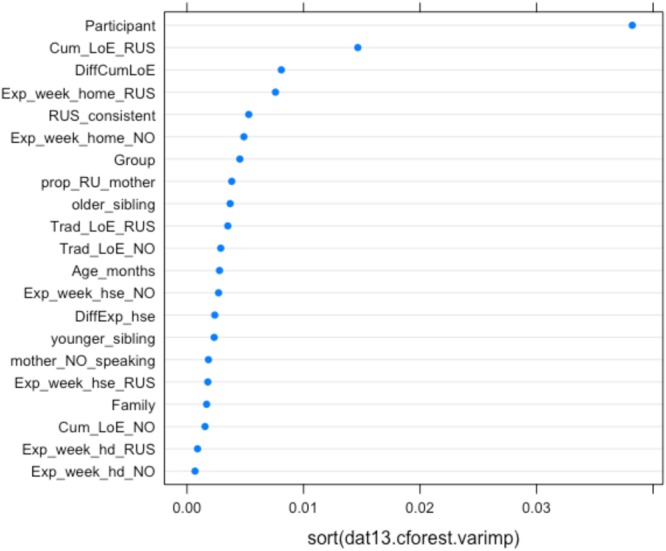
Conditional permutation variable importance for the random forest with all background predictors for children’s accuracy on two gender-assignment tasks. Predictors to the right of the 0.00 mark are significant.

**FIGURE 6:**
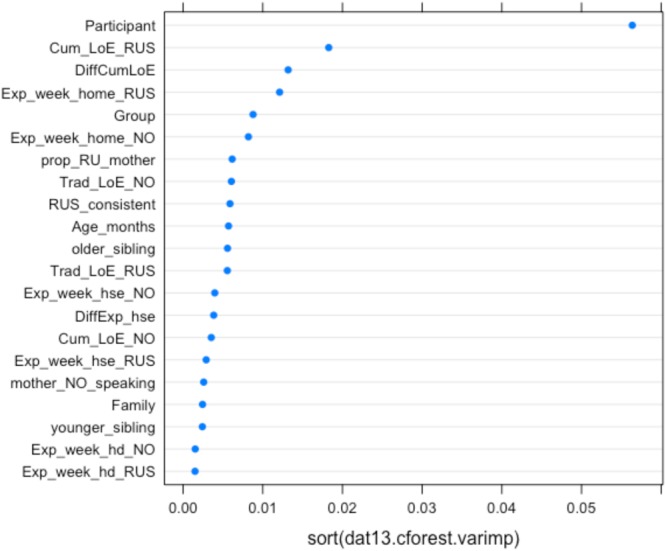
Conditional permutation variable importance for the random forest with all background predictors for the probability of masculine default on two gender-assignment tasks. Predictors to the right of the 0.00 mark are significant.

**FIGURE 7:**
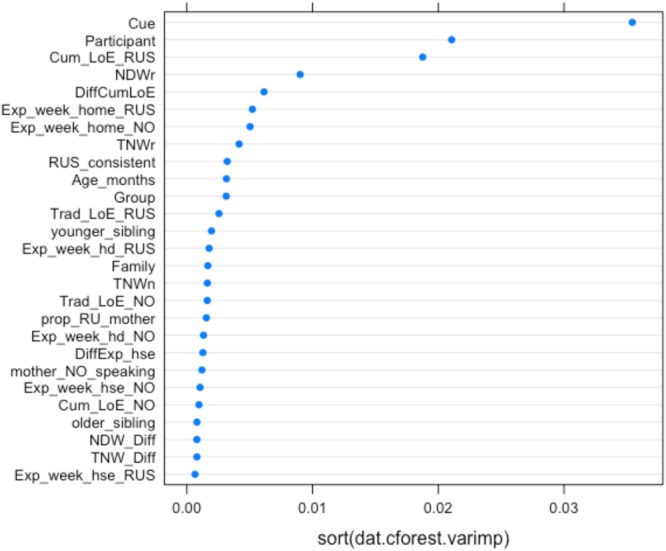
Conditional permutation variable importance for the random forest with all IVs (experimental, background and narrative) predicting HSs accuracy (for a subset of 27 children). Predictors to the right of the 0.00 mark are significant.

**FIGURE 8:**
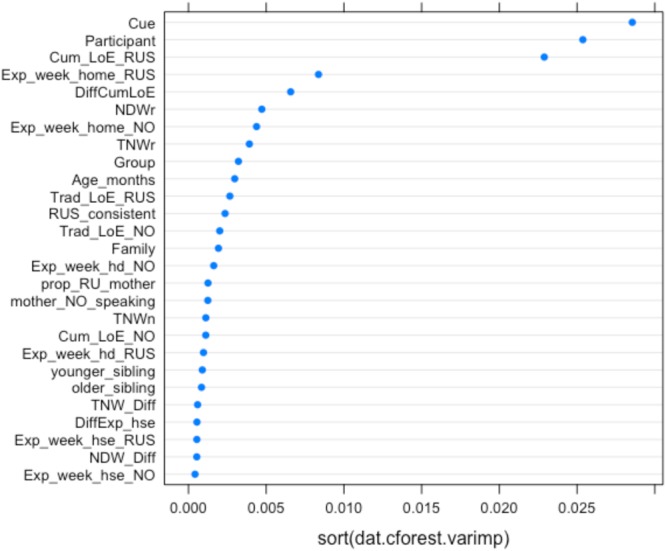
Conditional permutation variable importance for the random forest with all IVs (experimental, background, and narrative) in predicting the probability of masculine default (for a subset of 27 children). Predictors to the right of the 0.00 mark are significant.

One of the goals of our study was to estimate which of the background variables were the most robust and reliable predictors of the children’s performance on Russian gender assignment tasks. To do so, we applied a non-parametric approach (random forests analysis), in combination with standard generalized mixed effects linear regression modeling. We included 20 independent variables calculated with the help of BiLEC (Unsworth, 2013), which we had collected with the parents of the 54 bilingual participants (abbreviations used in the analysis and in **Figures 5–8** below are provided in the rightmost column):

**Table:** 

i.	Traditional length of exposure to Russian	Trad_LoE_RUS
ii.	Traditional length of exposure to Norwegian	Trad_LoE_NOR
iii.	Cumulative length of exposure to Russian	Cum_LoE_RUS
iv.	Cumulative length of exposure to Norwegian	Cum_LoE_NOR
v.	Weekly exposure to Russian at home (at present)	Exp_week_home_RUS
vi.	Weekly exposure to Norwegian at home (at present)	Exp_week_home_NOR
vii.	Weekly exposure to Russian at home, school and out-of-school activities (at present)	Exp_week_hse_RUS
viii.	Weekly exposure to Norwegian at home, school and out-of-school activities at present	Exp_week_hse_NOR
ix.	Consistency of input in Russian (Yes, if the mother^3^ indicated that she used Russian always or almost always with the child, No in all other cases)	RUS_consistent
x.	Proportion of Russian with mother	prop_RU_mother
xi.	Maternal proficiency in Norwegian	mother_NO_speaking
xii.	Age in months	Age_months
xiii.	Family type (NR vs. RR)	Family
xiv.	Group (daycare/school)	Group
xv.	Presence of older siblings	older_sibling
xvi.	Presence of younger siblings	younger_sibling
xvii.	Exposure to Russian during holidays (calculated as weekly exposure to Russian at home, school, extra and holidays at present minus weekly exposure to Russian at home, school and extra)	Exp_week_hd_RUS
xviii.	Exposure to Norwegian during holidays (calculated as weekly exposure to Norwegian at home, school, extra and holidays at present minus weekly exposure to Norwegian at home, school and extra)	Exp_week_hd_NO
xix.	Differences in current amount of exposure to Norwegian and Russian (calculated as Exposure to Russian at home, school and extra per week subtracted from Exposure to Norwegian at home, school and extra per week)	Diff_Exp_hse
xx.	Differences in cumulative amount of exposure to Norwegian and Russian (calculated as CLoE to Russian subtracted from CLoE to Norwegian)	DiffCumLoE

See **Table 3** for the descriptive statistics of the background variables.

**Table 3:** Background variables.

	All bilinguals	RR bilinguals	NR bilinguals	Russian L1
Number (*N* boys)	54 (27)	26 (15)	28 (13)	107 (67)
Age	4;0 – 10;2 (6;9)	4;0 – 10;2 (6;9)	4;3 – 9;9 (6;9)	3;0 – 7;0 (5;2)
	*SD* = 1.7	*SD* = 1.6	*SD* = 1.7	*SD* = 1.1
Group (daycare/school)	22/32	10/16	12/16	107/0
CLoE R (in years)	0.66 – 7.39 (3.48)	1.62 – 7.39 (4.68)	0.66 – 5.78 (2.39)	
	*SD* = 1.75	*SD* = 1.45	*SD* = 1.19	
CLoE N (in years)	0.33 – 7.03 (2.92)	0.33 – 3.18 (1.79)	1.49 – 7.03 (3.94)	
	*SD* = 1.61	*SD* = 0.67	*SD* = 1.52	
Diff CLoE (N-R)	–5.54 – 6.25 (-0.56)	–5.54 – 1.31 (-2.89)	–3.43 – 6.25 (1.56)	
	*SD* = 2.89	*SD* = 1.57	*SD* = 2.07	
Current exposure home/school/extra R (in %)	14 – 67 (42)	3 – 67 (54)	14 – 57 (30)	
	*SD* = 16	*SD* = 10	*SD* = 12	
Current exposure home/school/extra N	33 – 86 (58)	33 – 70 (46)	43 – 86 (69)	
	*SD* = 16	*SD* = 10	*SD* = 12	
Diff Current Exposure home/school/extra (N-R)	–34 – 72 (16)	–34 – 40 (-8)	–14 – 72 (38)	
	*SD* = 32	*SD* = 19	*SD* = 24	
Input consistency R (Y/N)	28/26	19/7	10/18	
Older sibling (Y/N)	28/26	14/12	12/16	
Younger sibling (Y/N)	18/36	8/18	10/18	

To assess the effect of the children’s background on their performance on the gender assignment tasks, we chose two binary dependent variables: accuracy and the probability of using masculine agreement in non-masculine conditions (masculine default). Note that in the opaque conditions of the nonce word experiment we coded both F and N responses in the FN condition and both F and M responses in the FM condition as ‘accurate.’

A serious challenge with data like ours has to do with the presence of many overlapping background variables. For example, exposure to Russian/Norwegian at home is collinear with exposure to Russian/Norwegian at home, school, and out-of-school activities; the amount of the child’s exposure to Russian at home is negatively correlated with their amount of exposure to Norwegian at home; Family type (NR vs. RR home) has a direct impact on the amount of input in Russian and Norwegian that the child receives at home; proportion of Russian with the mother inevitably correlates with other variables concerning input in Russian, etc.

One possible way to cope with multiple collinear predictors is to apply dimension reduction techniques, such as principal components analysis, and then use standard regression with the reduced set of variables (see e.g., Strobl et al., 2009). However, principal components analysis would only be appropriate for numeric variables and cannot be applied to variables of other types, e.g., factors, in our case: the presence/absence of older/younger siblings, consistent/inconsistent input in Russian etc. Furthermore, as argued in Strobl et al. (2009, p. 324), dimension reduction techniques have “the disadvantage that the original input variables are projected onto a reduced set of components, so that their individual effect is no longer identifiable.”

To overcome these limitations, we first ran a random forests analysis to estimate the relative importance of the different variables (see Breiman et al., 1984; Breiman, 2001; Strobl et al., 2009; Tagliamonte and Baayen, 2012). Random forests analysis is a non-parametric non-linear statistical method which makes it possible to analyze complex interactions between a large number of variables (Baayen, 2008). A random forest is a so-called “ensemble of classification or regression trees (CARTs), where each tree in the ensemble is built according to the principle of recursive partitioning, where the feature space is recursively split into regions containing observations with similar response values” (Strobl et al., 2009, p. 324). The advantages of this method include its applicability to data that are not normally distributed, as well as the fact that it allows for an automatic assessment of the relative importance of various variables in predicting the distribution of the data (cf., Tagliamonte and Baayen, 2012; Baayen et al., 2013).

However, as noted by Strobl et al. (2009), there are certain pitfalls connected to the fact that random forests were not developed in a stringent statistical framework, which might lead to potential confusion in the interpretation of main effects and interactions. To avoid these potential pitfalls, we decided to additionally run a standard mixed effects logistic regression analysis. We report the results of the models in turn and discuss the outcome of the analysis in the second part of the section.

#### I. Random Forests

We fit two random forests models^4^ (Hothorn et al., 2006; Strobl et al., 2008) to estimate the effect of 20 background variables (see above) on the children’s accuracy with respect to gender assignment (Model 1), and on the probability of making masculine default errors (Model 2). Note that models of this type do not differentiate between fixed and random effects; thus, we also included the variable Participant to estimate the variance attributed to individual differences.

**Figures 5, 6** depict the relative importance of the predictors, using conditional permutation-based variable importance (see Strobl et al., 2008). The variables presented in **Figures 5, 6** appear in accordance with their relative importance as predictors of the children’s accuracy (**Figure 5**) and probability of using masculine in non-masculine conditions (**Figure 6**). As the graphs show, the Participant is the most important predictor. This is not surprising, given that significant variability tied to the effect of individual participants is typical of psycholinguistic research in general (see e.g., Baayen, 2008; Tagliamonte and Baayen, 2012). The next most important predictor is CLoE to Russian, which is considerably more important than all other background variables. Significant predictivity is also detectable for Exposure to Russian and Norwegian at home per week (at present), Consistency of Russian input, Proportion of Russian with mother, Traditional length of exposure to Russian, Group, Presence of an older sibling, followed by the remaining variables. Note that Family type is generally ranked low in the hierarchy of predictors, suggesting that although the effect of Family type is significant, other variables have a much larger predictive power. In the next section, we present the analysis couched within the generalized linear mixed model approach.

#### II. Generalized Linear Mixed Models

Recall that the reasons for including mixed effects logistic regressions were the following: (1) to assess the significance of the variables using a stringent statistical framework; (2) to assess whether the correlation between the variables is positive or negative; (3) to check for collinearity of fixed effects, and (4) to include random effects of Items and Participants (note that in the random forests approach, random and fixed effects are not distinguished). In the logistic regressions, we included the ten most important variables from the random forests analysis and used them as predictors (apart from Participant, which was included as a random effect).

Model 1: Accuracy as predicted by the child’s background. The following variables correlated significantly with the children’s accuracy:

(i) CLoE to Russian (positive correlation *p* < 0.001: children with a higher cumulative exposure index were more likely to assign gender accurately);
(ii) Consistency of input in Russian (positive correlation *p* < 0.01: children who received consistent input in Russian were more accurate)
(iii) The presence of an older sibling (negative correlation *p* < 0.001: children who had an older sibling were overall less accurate than those who did not).
(iv) Group (positive correlation *p* < 0.05: schoolchildren were more accurate than daycare children).

Model 2: Defaulting to masculine agreement (in non-masculine conditions) as predicted by the child’s background. The probability of using masculine as the default correlated significantly with the following predictors:

(i) CLoE to Russian (negative correlation *p* < 0.001: children with a higher cumulative exposure index were less likely to default to masculine);
(ii) Consistency of input in Russian (negative correlation *p* < 0.05: children who received consistent input in Russian were less likely to default to masculine);
(iii) The presence of an older sibling (positive correlation *p* < 0.001: children who had an older sibling were more likely to make masculine default errors).

### Narrative Proficiency Measures

We collected child narratives in Norwegian and Russian with a subset of 27 out of the 54 participants. This group of 27 children is representative of the whole set of bilingual participants both in terms of family type (14 of the children were from NR homes and 13 from RR homes) and in terms of age (age range 4;0–9;6, mean age 6;8), which is comparable with the distribution in the whole sample.

Based on the children’s narratives we calculated the following four variables: Total number of words in the Russian narrative (TNW), Total number of words in the Norwegian narrative (TNWn), Number of different words in the Russian narrative (NDW), and Number of different words in the Norwegian narrative (NDWn). In the analysis, we also included two relative variables: the difference between NDW in Norwegian and Russian, and the difference between the TNW in Norwegian and Russian (see **Table 4** for the descriptive statistics of the narrative variables). As evident from **Table 4**, the relative variables are mostly positive, suggesting that the majority of the participants used more words overall, as well as more different lexical words, in the Norwegian narratives than in the Russian narratives (only two children had slightly higher NDW and TNW scores in Russian than in Norwegian).

**Table 4:** Narrative variables.

	All bilinguals	RR bilinguals	NR bilinguals
NDW R	4 – 53 (26)	21 – 53 (31)	4 – 37 (21)
	*SD* = 10	*SD* = 8.9	*SD* = 8.8
NDW N	27 – 74 (42)	30 – 63(43)	27 – 74 (41)
	*SD* = 10	*SD* = 10	*SD* = 11
Diff NDW (N-R)	–12 – 41 (16)	–12 – 32 (12)	4 – 41 (20)
	*SD* = 11	*SD* = 12	*SD* = 8
TNW R	8 – 88 (42)	32 – 88 (51)	8 – 54 (34)
	*SD* = 17	*SD* = 16	*SD* = 13
TNW N	47 – 180 (90)	54 – 126 (89)	47 – 180 (90)
	*SD* = 29	*SD* = 23	*SD* = 34
Diff TNW (N-R)	–1 – 137 (48)	–1 – 73 (38)	32 – 137 (56)
	*SD* = 27	*SD* = 23	*SD* = 28

Based on these measures we conducted a combined analysis of the data from the 27 bilingual children which included all variables: experimental variable (Condition), individual-level variable Participant, and all background and narrative variables.

**Figures 7, 8** show the relative importance of the predictors in explaining accuracy (**Figure 7**) and probability of defaulting to masculine (**Figure 8**).

As **Figures 7, 8** show, the four most important predictors are Participant, Condition (Cue), CLoE to Russian, and NDW in the Russian narrative (our lexical diversity measure in the heritage language). Other background and narrative predictors are ranked below these variables.

To test the effects of the predictors in a more stringent statistical approach, we fit a set of generalized linear mixed effects logistic regression models to predict accuracy (Models 1a and 2a) and probability of making masculine default errors (Models 1b and 2b) with a combined set of experimental, background, and narrative predictors. Models 1a and 1b included the following main effects: an experimental variable (Condition) and three BiLEC variables (CLoE to Russian, presence of an older sibling, and weekly exposure to Norwegian, i.e., the variables which were ranked highest in predicting masculine default errors in the random forests analysis). Models 2a and 2b additionally included two narrative variables (NDW in the Russian and Norwegian narratives). We excluded other narrative variables to avoid collinearity and achieve model convergence. Participants and Items were taken as random effects in both models. The aim of this analysis was to establish whether the inclusion of the narrative variables would significantly improve model fit, or whether the narrative variables can be safely disregarded.

The two main predictors: CLoE to Russian and NDW in the Russian narrative both correlated positively with the children’s accuracy (*p* < 0.05 and *p* < 0.01, respectively) and negatively with the probability of masculine default errors (*p* < 0.05 for both correlations). NDW in the Norwegian narrative was not a significant predictor.

A likelihood ratio test (ANOVA) of Models 1a and 2a as well as Models 1b and 2b showed that the bigger models (which additionally included predictors from the narrative tasks) should be preferred, despite the higher number of predictors involved. Models 2a and 2b were significantly better than their Model 1 counterparts (*p* < 0.001 in both cases).

To sum up, converging results from parametric and non-parametric statistical modeling show that the number of different words (NDW) used in the Russian narrative task and the CLoE to Russian (CumLoE) are both reliable predictors of children’s grammatical development in their heritage language, illustrated by the acquisition of gender assignment patterns in this study. Furthermore, these predictors do not overlap, but complement each other, and the inclusion of the narrative measure significantly improves model fit and explains more variance in the data.

Finally, we also tested whether the inclusion of the dominance variable (differences in the cumulative amount of exposure between Norwegian and Russian) that was ranked high in the random forests analysis could significantly improve the model fit. We fit two additional generalized linear mixed effects regression models, Models 3a and 3b, which included the same predictors as Models 2a and 2b, but additionally a variable reflecting Difference in CLoE between Norwegian and Russian. A likelihood ratio test (ANOVA) of Models 2a and 3a and Models 2b and 3b showed that the bigger models (which additionally include the dominance predictor) do not survive the comparison. The difference between the models was not significant, and the criteria for model selection (Bayesian information criterion and Akaike information criterion) were smaller for Models 2a and 2b, which is preferable. This means that adding the relative dominance variable to the model does not improve model fit and does not explain any additional part of the variance that is not covered by the absolute exposure and proficiency variables, such as CLoE and lexical diversity in the heritage language.

## Discussion

### Gender Knowledge and Cue Sensitivity

With regard to gender knowledge and cue sensitivity of bilinguals we asked the following two main questions:

(1) Do heritage speakers of Russian (HSs) differ significantly from monolinguals and in what conditions (on the real and nonce word tasks)?
(2) Are there any differences between nouns with transparent and nouns with non-transparent morphophonological gender cues, for both monolinguals and HSs (on the real and nonce word tasks?

In the real word task, no differences between groups were found in the masculine conditions, with RR, NR and monolingual children all performing at ceiling. However, with masculine being considered the default gender, target-like performance in the masculine condition does not allow us to disambiguate between actual internalized knowledge of the grammatical gender of a given item and a defaulting strategy. In the feminine and neuter conditions, the statistical analysis revealed that the NR children were significantly less target-like than monolingual controls, while no statistical difference was found between the monolinguals and the RR children. Overuse of masculine in non-masculine conditions was found in both bilingual groups, but it was most prevalent in the data of the NR bilinguals. In the case of vowel-final items (feminine transparent, neuter transparent, neuter opaque), these results are different from those of Polinsky (2008), who finds that vowel-final items are overgeneralized to the feminine and consonant-final items to the masculine in adult heritage speakers. In other words, Polinsky (2008) finds evidence for a restructured (and simplified) grammatical gender system, where the binary opposition in grammatical gender (masculine vs. feminine) is marked by means of a binary phonological contrast (consonants vs. vowels).

Notably, our participants and the participants in Polinsky’s study differ in at least two important respects: age and majority language. The participants in Polinsky’s study are adults who were born and raised in Russian immigrant families in the United States. Our participants were children born and raised in Russian-Russian and Norwegian-Russian families in Norway. Given significant age differences between the groups, we cannot rule out the possibility that the two distinct patterns (defaulting to masculine across the board vs. restructuring of the 3-gender system into a simplified masculine vs. feminine system) might reflect two different stages of heritage language development (cf. Polinsky, 2016). For instance, it may be the case that with more exposure to Russian, feminine and neuter genders will be acquired by some of our participants and a three-gender system will be developed. However, it may also be the case that some of the children will never acquire the target grammatical gender distinctions, and hence this can be considered a case of incomplete acquisition (in fact, we believe that for two of our participants who are already above the age of 8 but have produced exclusively masculine agreement in both gender tasks and narratives, this might be the case). The participants in Polinsky (2008) study may on the other hand already show signs of attrition – it is possible that they had had a three-gender system at some point of their grammatical development (e.g., at the pre-school age), but later developed a simplified two-gender system due to attrition and lack of contact with Russian.

Cross-linguistic influence from the majority language may also be a factor contributing to the observed difference between the patterns reported in Polinsky (2008) and in our study. Masculine is without doubt the morphological default in Norwegian, with feminine being the most vulnerable gender. In many dialects, feminine is disappearing and is being replaced by masculine (Lødrup, 2011; Rodina and Westergaard, 2015; Busterud et al., forthcoming). It is conceivable that the role of masculine in heritage Russian is strengthened under the influence of Norwegian, a language with a strong masculine default. Potential supporting evidence for this idea comes from a study concerning gender acquisition in Russian by Russian-German bilingual children (Dieser, 2009). This study found that Russian-German children, especially those with a small CLoE to Russian and a low amount of different words in Russian narratives, tended to default to feminine, which has been argued to be the default gender in child German (Kupisch et al., 2018).

Interestingly, we do find that RR children overgeneralize neuters to the feminine as well as to masculine (and the trend persists with nonce items as well). This might be taken as evidence that RR children are more likely to assign nouns to gender categories based on (some generalizations over) morphophonological cues, while NR children simply rely on the default. In other words, the RR children (as well as the monolinguals) seem to be more sensitive to the fact that there is a phonological similarity between final-unstressed neuters and final-unstressed feminines, and their mis-assignment of the former stems from this knowledge. In contrast, NR children prevalently overgeneralized final-unstressed neuters to the masculine, which suggests that they are oblivious to this similarity.

Finally, the transparency of gender cues only played a significant role in the feminine conditions, with all groups of participants showing higher accuracy with transparent feminine items. It is likely that the distinction between transparent and opaque masculines may be masked by the masculine default strategy. The difference between transparent and opaque neuters did not surface in the case of bilinguals, because neuters were generally a challenge for them due to their low input frequency.

Turning now to the nonce word task, it was found that both RR and NR bilinguals were significantly less target-appropriate than monolingual controls on the feminine transparent and neuter transparent nouns. The difference in accuracy between the two bilingual groups reached statistical significance in the feminine transparent condition, although the NR bilinguals gave more non-target appropriate responses than the RR bilinguals in the neuter transparent condition as well. The RR bilinguals patterned with monolinguals in that they preferred feminine in the ambiguous FN and FM conditions, while the NR bilinguals used significantly more masculine agreement in both conditions. Overall, the results show that purely cue-based gender assignment is more challenging for the bilinguals, while the differences between the bilingual groups indicate that the amount of exposure plays a role. At the same time, it needs to be stressed that all groups of participants showed sensitivity to phonological gender cues – albeit to different degrees. Neuter responses were given exclusively in neuter conditions, and the proportion of feminine responses was significantly higher in those conditions where it is target-appropriate.

Our research questions 3 and 4 addressed the role of lexical learning. Specifically, we asked whether bilinguals rely on lexically stored gender features and whether this behavior may be more pronounced in HSs than in monolinguals. Our results show that accuracy across the three transparent conditions on the real word task was significantly higher than on the nonce word task for all three groups of participants, with the interaction of group and task being not significant. This might be taken as evidence that lexical learning of the gender category of familiar nouns in addition to cue-based assignment is an important strategy in grammatical gender acquisition for both bilinguals and monolinguals. Additionally, the difference between real and nonce items may be attributed to the difference in the cognitive load required by each task. It is reasonable to assume that when the grammatical gender of a noun is acquired (regardless of whether it was deduced from noun-internal cues or distributional information) it is stored in the lexical entry and retrieved as needed rather than computed online each time the lexical item is invoked (Caramazza et al., 1988). On-the-go gender assignment of a novel noun, on the other hand, presupposes online computation, which is arguably a more cognitively demanding process than retrieval and therefore more error-prone.

### The Role of Exposure, Proficiency, and Dominance

Research questions 5–8 investigated to what extent a composite measure of proficiency and amount of exposure influenced heritage speakers’ performance on gender assignment tasks. Specifically, we asked which of the background variables were the most reliable predictors of performance on the gender assignment tasks, whether lexical measures of bilinguals’ performance in the narrative task would correlate significantly with their performance on the gender assignment tasks, and whether the use of a composite measure of background data and narrative proficiency would have an advantage in predicting the performance of HSs on the gender assignment task (as compared to using only background or only proficiency measures).

Question 5 asked which of the 20 background variables were the most reliable predictors of bilinguals’ performance on the gender assignment tasks. The results showed that CLoE to Russian was by far the most reliable and predictive variable that accounted for the largest portion of the variance in the data. A number of other variables, such as Consistency of input in Russian, Traditional length of exposure to Russian, Proportion of Russian with the Russian-speaking parent (mother), as well as Presence/absence of an older sibling were also high in the hierarchy of the most important predictors (see **Figures 5, 6**). Statistical significance of CLoE to Russian (positive correlation with accuracy, negative correlation with the probability of defaulting to M) as well as a number of other background variables (see above) was confirmed through subsequent generalized linear mixed effects regression analysis. To sum up, converging results of parametric and non-parametric statistical models indicate that CLoE to Russian is the most robust and reliable background variable that can be taken as a proxy of the children’s amount of exposure to Russian. In other words, our results show that CLoE to Russian is a better predictor of heritage speakers’ level of acquisition of grammatical gender in Russian than other background variables.

Questions 6 and 7 asked whether the lexical measures of proficiency obtained from the narrative samples in both languages correlated with HSs’ performance on the gender assignment tasks. Our analysis included four variables: Total number of words (TNW) in the Russian narrative, TNW in the Norwegian narrative, Number of different words (NDW) in the Russian narrative, and NDW in the Norwegian narrative. NDW in the Russian narrative was ranked highest of all narrative variables in the random forest analysis. Subsequent generalized mixed logistic regression analysis confirmed that out of all narrative variables, only NDW in the Russian narrative correlated significantly with accuracy (positive correlation) and with the probability of masculine default errors (negative correlation).

Multiple recent studies have suggested that combining various language proficiency measures (production, comprehension, repetition, etc.) with background measures quantifying language exposure and use would be fruitful in modeling heritage speakers’ grammatical abilities (Montrul, 2015, 2016, chapter 6; Polinsky, 2018: chapter 3). We included two types of variables into our analysis and showed that background measures and narrative proficiency measures are both significant predictors of children’s performance on gender assignment tasks. Furthermore, we showed that combining background measures with narrative proficiency measures improved the predictive power of the statistical model. This indicates that narrative proficiency measures in the heritage language have an independent value as predictors of HSs’ acquisition of grammatical gender, in addition to language exposure variables.

Our last question addressed the independent effect of language dominance – in addition to absolute background and narrative proficiency measures – on the HSs’ performance with respect to gender assignment. We used three variables to quantify dominance: (i) the difference in the CLoE between the majority and the minority languages, (ii) the difference in current exposure to the two languages, and (iii) the difference in the relative scores on the narrative tasks in the two languages. The only variable that was ranked relatively high on the variable importance hierarchy in the random forests analysis – although still below CLoE to Russian – was the difference in the cumulative exposure between the two languages. However, the results of a model comparison showed that the inclusion of this variable in addition to CLoE to Russian did not improve model fit. It is thus likely that the importance of this variable might be an artifact of collinearity between this variable and CLoE to Russian: the more Russian input the children accumulate, the smaller the difference between cumulative exposure to Norwegian and to Russian. None of the dominance variables turned out to have an independent value for our analysis, since all the variation they accounted for could be captured by variables that measure absolute cumulative exposure and proficiency in the heritage language.

## Conclusion

In this paper, we have shown that a combination of background variables and proficiency measures predicts heritage speakers’ performance on grammatical gender tasks in Russian better than background measures or narrative proficiency measures taken in isolation. We carried out two production experiments investigating gender assignment to real as well as nonce words in Russian, including all three genders and transparent as well as opaque cues. Participants were 54 Norwegian-Russian bilingual children living in Norway (age range 4;0–10;2) and 107 monolingual controls. Background information was collected through the *Bilingual Language Exposure Calculator* (Unsworth, 2013), and the proficiency measure was based on the MAIN semi-spontaneous narratives (Gagarina et al., 2012). As many as 20 background variables and six proficiency measures were included in the statistical analysis of the participants’ performance on the gender tasks. We also included three dominance variables (the difference in CLoE between the majority and the heritage language, the difference in current exposure to the two languages, and the difference in scores on Russian and Norwegian narratives). The best predictors turned out to be a combination of three background variables (CLoE to Russian, consistency of input, and the presence of older siblings) and one proficiency measure, lexical diversity as defined by the number of different words in the Russian narrative. Interestingly, our statistical analysis showed that the dominance variables are not robust predictors for the bilingual children’s performance on gender assignment. We argue that these results support Montrul’s (2015) distinction between dominance and proficiency: Language dominance vs. non-dominance is a relative concept and may reflect considerable variation with respect to proficiency in the heritage language.

## Ethics Statement

The project was registered and approved by the Norwegian Social Science Data Service (NSD, http://www.nsd.uib.no). Data collection was conducted in accordance with NSD’s ethical principles. Written informed consent was obtained from parents of all the participants prior to testing.

## Author Contributions

All co-authors are responsible for the conception of the work and experimental design. NM carried out the collection of control data with monolingual Russian children. NM, YR, and OU carried out the collection of data with bilingual participants. MW collected Norwegian narrative data with bilingual participants. NM is responsible for data analysis and interpretation of the results. All authors share responsibility for drafting of the work and final approval of the version to be published.

## Conflict of Interest Statement

The authors declare that the research was conducted in the absence of any commercial or financial relationships that could be construed as a potential conflict of interest.

## Footnotes

1 All models were fit using R version 3.4.4 (release 2018-03-15)

2 This was a rather unexpected result, which can be attributed to the fact that a small group of 3-year-old monolinguals defaulted to feminine across the board in this task, thus lowering the overall proportion of M in this condition, while defaulting to F was not observed in the bilingual groups.

3 All NR children in our sample had a Russian-speaking mother and a Norwegian-speaking father. The fathers reported no or low proficiency in Russian. In the analysis, we included three “maternal” variables: consistency and proportion of Russian input (with mother), as well as maternal proficiency in Norwegian (as it might be expected that that the more fluent the mother is in Norwegian, the more likely she would be to use Norwegian at home with other family members and her children).

4 The function *cforest* of the R package *party* was used for the analysis of variable importance (Breiman et al., 2006; cf. Strobl et al., 2008).
